# Cases in Practice

**Published:** 1874-07

**Authors:** F. K. Bailey

**Affiliations:** Knoxville, Tenn.


### CASES IN PRACTICE.

BY F. K. BAILEY, M.D., KNOXVILLE, TENN.

### *I. MARASMUS WITH FACIAL PARALYSIS.*

About 1st February, 1874, I was called to see a female child, of negro parentage, fifteen months old, which, for some weeks past has had a wasting looseness of the bowels; emanciated, especially in the lower limbs; abdomen somewhat full, but not distended; but little appetite; takes the breast. Prescribed tr. muriate of iron with chlorate of potash, and Dover's powder at night to quiet restlessness.

February 8th.—Improving rapidly; bowels not loose, and appetite improving; takes food with avidity. To continue iron and chlorate of potass.

February 15th.—Has high fever, with gastric disturbance; abdomen distended. Suspect worms. Gave calomel and santonine, to be followed with castor oil.

February 17th.—Much improved. No indications of worms in the stools. To continue the iron mixture.

February 19th.—Called and found the child improving in its general condition, with a desire for food, but the left side of the face is drawn in, and the angle of the mouth drawn back and a little downwards. No driveling of saliva, but some difficulty in chewing and controlling a morsel of food. First noticed on yesterday, and last night there was want of uniformity in the closure of the left eyelid, but none noticeable to-day.

I will here remark that for some days past there has been a free discharge of pus from the ears, more particularly from the left side. The ears had been freely wiped out with a solution of carbolic acid and hyposulphite of soda.

Still continue the iron mixture and lotion for the ears.

February 22d.—Found the child somewhat improved in general appearance. When the face is at rest, there is but little difference to be seen in the two sides, but on its crying, the whole cheek is drawn back, and the angle of the mouth depressed. The ears discharge less; the bowels are not loose, and the strength gaining. much more desirous for food. To continue tr. mur. iron mixture, with sulph. cinchoniae added.

March 23d.—Had not seen the child till to-day, when the mother brought her to my office, and states that during the past month the bowels have not been loose, and the appetite has been good. Appears to have grown in height and can creep about upon the floor, and draw itself up by a chair, which indicate nutrition and increasing strength.

But there is no increase of tissue in the lower extremities, which are spindle shaped. There has been some cough of late, and I find a rale in both lungs—worse in the left. No change in the facial appearances. The angle of the mouth on the left side is drawn backwards, and rather upwards, with a sunken appearance to the cheek. Discharge from the left ear very free, with some from the right. To continue the lotion for the ears, and increase the dose of the iron mixture.

March 27th.—No improvement since the twenty-third. Will sit up for hours in a chair, but is unwilling to lie down during the day. Continue treatment.

April 8th.—This child died about the first instant, sinking away gradually, but had some convulsive threatenings at last. I attribute the facial paralysis to some pressure upon the nerves caused by the disease in the ears. The immediate cause of death was cerebral probably.

This case is interesting on account of the paralysis, although the marasmic developments are not devoid of interest to the pathologist. It is evident that recovery might have resulted, were it not for the nervous complications.

### *II. CARDIAC PHENOMINA FROM REFLEX INFLUENCES.*

Betty M., aged ten, mulatto. Taken April 17th, 1874, with a pain in the left side of the chest, extending to the shoulder and down to the middle of the arm. I was called on the 20th and found the tongue covered the whole extent with a white coat, thick and creamy; pulse 110, small and quick; bowels have been constipated, but open this morning.

On applying the ear to the cardiac region, find increased impulse; slight friction sound, and a diastolic flapping towards the apex; some bronchial rale in the left side; urine, scanty, thick and of a yellowish hue for some days; has been rheumatic during the winter.

Prescribed 4 gr. doses of pulv. doveri, every four hours till three are taken, and the following mixture in teaspoonful doses four times daily: ℞. Iodidi potassii, ʒss; bromidi potassii, ʒj; bicarb. potassæ, ʒj; syr. scillæ, ℥ss; syr. solutani, ℥iss. M. To remain quiet in bed, and have light diet.

April 21st, 5 p. m.—Much better; no pain; free from fever last night; pulse natural; bowels open. To continue mixture, with pulv. doveri at night.

April 23d.—Sitting up in a chair; some cardiac pain, but less impulse; flapping diminishing in distinctness. Continue mixture, and to take a powder of calomel and santonin at night.

May 2d.—Betty passed a number of round worms after my last visit; in and about the streets.

In this case is seen an instance of a conditon caused by a rheumatic diathesis, together with a rather feble organization.

There is some cardiac trouble at her best estate, and the damp, cool spring weather has kept up a want of action in the skin, and induced a corresponding determination to internal organs. With a good sprinkling of Anglo-Saxon blood, she is of a tender organization, destined to sink at an early age under the manifold ills her race is liable to. Under favorable circumstances she *may* pass the age of puberty and become a young woman of some strength.

Both possibilities and probabilities are against her living to the age of eighteen

Worms are common in children here of both black and white parentage, unless well nourished and sheltered. On the other hand, among our well-to-do white families, it is rare that the large round worms is ever seen. Colored girls who have any white blood in them, are apt to become anemic and victims to cardiac disease in various forms.

Nothing but tonics and generous diet will avert such a contingency, and these children seldom have proper medicines or a sufficiency of food.

The worms which generally are a result of the want of proper care, will in their turn prove a source of reflex action upon the nervous centres and circulatory system, and add to the unhappy train of influences.

